# Low Memory Access Video Stabilization for Low-Cost Camera SoC

**DOI:** 10.3390/s22062341

**Published:** 2022-03-18

**Authors:** Yun-Gu Lee

**Affiliations:** School of Software, Kwangwoon University, Kwangwoon-ro 20, Nowon-gu, Seoul 01897, Korea; harmony96@gmail.com; Tel.: +82-2-940-8112

**Keywords:** video stabilization, rolling shutter compensation, CMOS sensor, camera SoC, camera hardware

## Abstract

Video stabilization is one of the most important features in consumer cameras. Even simple video stabilization algorithms may need to access the frames several times to generate a stabilized output image, which places a significant burden on the camera hardware. This high-memory-access requirement makes it difficult to implement video stabilization in real time on low-cost camera SoC. Reduction of the memory usage is a critical issue in camera hardware. This paper presents a structure and layout method to efficiently implement video stabilization for low-end hardware devices in terms of shared memory access amount. The proposed method places sub-components of video stabilization in a parasitic form in other processing blocks, and the sub-components reuse data read from other processing blocks without directly accessing data in the shared memory. Although the proposed method is not superior to the state-of-the-art methods applied in post-processing in terms of video quality, it provides sufficient performance to lower the cost of camera hardware for the development of real-time devices. According to my analysis, the proposed one reduces the memory access amount by 21.1 times compared to the straightforward method.

## 1. Introduction

**Figure 1:**
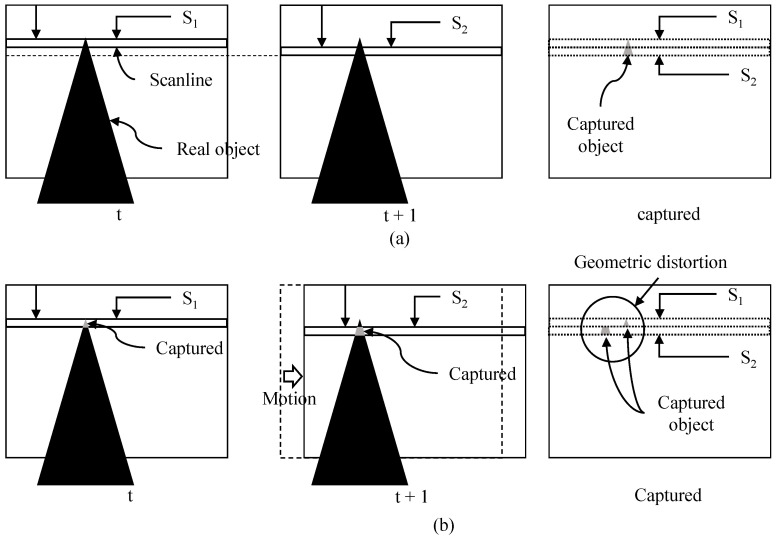
(**a**) Readout circuits read the pixel values from top to bottom. (**b**) Geometric image distortion from rolling shutter if camera moves.

**Figure 2:**
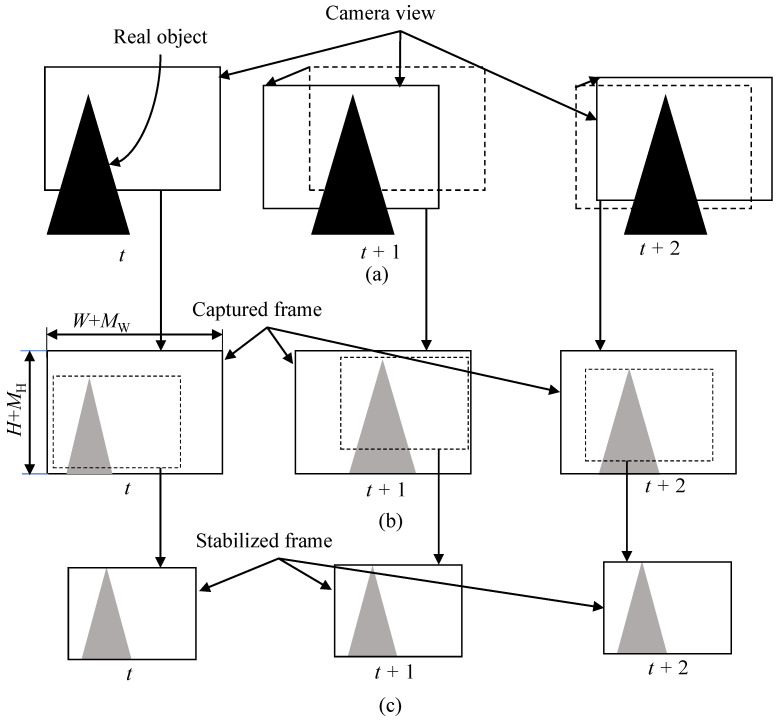
(**a**) Camera motion along time and (**b**) unstable video (captured). (**c**) Captured frames are cropped to generate stabilized output frames.

Nowadays, various types of devices from low-end to high-end are equipped with a camera that adopts a complementary metal-oxide semiconductor (CMOS) sensor [1]. CMOS technology provides low power consumption and fast readout speed [2], but the quality of video shot by the camera is degraded when the camera moves during capturing a video [3]. The first degradation is the undesirable geometric image distortion that comes from the rolling shutter effect [4]. As depicted in Figure 1, readout circuits of the CMOS sensors read the pixel values row by row from top to bottom rather than all the rows at the same time, which is called rolling shutter [4,5]. The CMOS sensor captures each row in a frame at the different time due to the rolling shutter effect [6]. For example, rows of $S_{1}$ and $S_{2}$ in Figure 1 are captured at the different time. Readout circuits first read the pixel values at row $S_{1}$, then the pixel values at row $S_{2}$ are captured. If a camera moves from time *t* to t+1 as given in Figure 1b, each row will have different amount of displacement according to camera motion. Thus, the captured image will be distorted as in the right image of in Figure 1b. This geometric distortion causes a straight line to bend. The second cause of quality degradation is camera motion jitters along frames, as depicted in Figure 2. Camera motion along frames in Figure 2a makes a captured video shaky, as depicted in Figure 2b. These two factors significantly degrade the quality of video, and users are uncomfortable watching the video. Hence, video stabilization with rolling shutter compensation is one of the most important features in consumer cameras.

Many algorithms have been developed to compensate the geometric distortion from the rolling shutter effect and stabilize the shaky video [6,7,8,9,10,11,12,13,14,15,16,17,18,19]. A simple two-dimensional model was proposed to remove perceived camera motion [7]. Liang [6] proposed a method to use planar motion for modeling the rolling shutter effect. A content-preserving warp was proposed by relaxing the physical constraint while preserving the perceptual quality [9]. Liu proposed video stabilization using on subspace constraints on the 2D feature trajectories [13]. Forssen and Ringaby proposed a method to use feature point matching and tracking for video stabilization and rolling shutter compensation [11,14]. Grundmann proposed calibration-free video stabilization and rolling shutter compensation based on a mixture model of homographies [15]. Lee proposed video stabilization based on the human visual system [16]. Guilluy recently reviewed more than 120 articles related to digital video stabilization [20] in depth in terms of cutting edges and gaps. Camera motion basically causes image quality degradation. Therefore, many existing algorithms have been focused on improving the camera motion models that describe the geometric relationship among consecutive frames [20]. Recently, CNN-based methods [21,22,23] have been studied, but they require high computational resources [20]. While these algorithms provide nice performance, they require huge computational burden and are suitable for use with post-processing [18]. Dong [18] hence proposed fast video stabilization for strict real-time applications and dramatically reduced the processing complexity. However, it is not still suitable for use with real-time mobile applications [19]. To further reduce the processing time, real-time method for a CMOS image sensor was proposed [2,19]. Its computational complexity is extremely low enough to run it on CPUs in from middle-end to high-end mobile devices. However, the above software-based algorithm may not be possible to run on CPUs in low-end devices such as cheap drones, cameras, IP cameras, etc. The computational resources of these low-end devices are so limited that there is not room enough to allocate the computing resources for video stabilization with rolling shutter compensation (VSRSC).

For real-time processing in low-end devices, we can consider hardware implementation on camera system-on-chip (SoC). Compared to software-based algorithms, hardware-based algorithms have not been studied much. Araneda proposed a hardware architecture for real-time digital video stabilization [24] and a compact hardware architecture for digital image stabilization using integral projection [25]. However, these architectures did not consider the rolling shutter compensation that is one of the most important features in cameras equipped with the CMOS sensor. There are two major concerns in implementing the algorithm using hardware: hardware complexity increase and additional memory access. The hardware complexity may be significantly reduced by adopting fast algorithms such as [2,19]. However, VSRSC requires very large additional memory access, which is a huge burden in real-time devices, especially for low-end. The reduction of memory bandwidth is always a critical issue in camera SoC [26], and study on video stabilization and rolling shutter compensation that uses small amount of memory access is needed. Let us consider the memory bandwidth for running VSRSC in brief. VSRSC generally consists of three parts: motion estimation, new camera path planning (or parameter estimation for compensation), and new image synthesis [2]. Motion estimation predicts camera motion by analyzing successive frames. Thus, it needs to read two successive frames at least from memory and compare them to predict motion. VSRSC usually needs local motion to predict the geometric distortion from rolling shutter compensation. It makes the memory access pattern very irregular or random and significantly decreases the efficiency of the system bus. In general, the local motion estimation requires more than two-frame memory access. In addition, even simple image warping should read a frame from the shared memory, synthesize the new frame, and write the frame to the shared memory. In summary, VSRSC may require three frames reading and one frame writing at minimum. A dedicated internal memory may be used to reduce memory access of the shared memory. However, the dedicated internal memory to store frames is too expensive for low-end camera SoC. It is necessary for camera hardware to efficiently reduce the memory bandwidth required for the processing while providing acceptable performance.

One of the biggest challenges in VSRSC for low-end devices is to reduce memory access amount while providing acceptable performance. Although Araneda proposed a solution for real-time digital video stabilization [24], it did not consider the reduction of the memory access amount. Moreover, its image quality may not be acceptable without the rolling shutter compensation. This paper proposes a structure and layout method to efficiently implement our previous real-time algorithm [2] for low-end hardware devices in terms of shared memory access amount. The memory access amount varies depending on the structure in which each sub-component of the algorithm is arranged and structured. Sub-components should be arranged and structured so that data read from the shared memory can be reused as much as possible. This paper presents a method where sub-component block for VSRSC reuses data read by other processing blocks. For this purpose, this paper analyzes the memory access pattern of sub-components in our previous VSRSC, and each sub-component is placed in a parasitic form in other processing blocks in consideration of the memory access pattern. Consequently, the memory access amount is extremely reduced.

## 2. Review

This paper analyzes the computational operations and memory access patterns of our previous software-based algorithm [2], and arranges sub-components of the algorithm in camera SoC so that shared memory access is reduced when implementing it in hardware. Therefore, this section briefly reviews our previous rolling shutter compensating algorithm from a computational point of view.

### 2.1. Motion Estimation

A line motion vector at each row is required to compensate the geometric distortion from rolling shutter effect. Our previous method obtains the line motion vector at each row by not directly predicting motion but mathematically deriving it from several global motion vectors. Since the implementation cost of global motion estimation is cheaper than that of local motion estimation, this approach significantly reduces the implementation complexity. Well-known projection-based motion estimation is adopted to further reduce implementation cost of the global motion estimation [27]. The projected-based global motion estimation converts two-dimensional motion search to one-dimensional motion search. Frames are first projected to create one-dimensional lines along the horizontal and vertical directions. Let the projected lines be $L_{n}^{X}\left(x\right)$ and $L_{n}^{Y}\left(y\right)$.

(1) $$\begin{array}{c} L_{n}^{X}\left(x\right)=\sum_{j=0}^{H-1}I_{n}\left(x,j\right)\;\;\;\;\;L_{n}^{Y}\left(y\right)=\sum_{i=0}^{W-1}I_{n}\left(i,y\right) \end{array}$$

Here, $I_{n}\left(i,j\right)$ is an intensity of pixel at (*i*, *j*) of the *n*-th frame. Then, global motion is predicted using the projected one-dimensional lines as follows.

(2) $$\begin{array}{c} GMV^{X}\left(n\right)=\arg\min_{m\in [-S,S]}\sum_{i=S}^{W-1-S}\left|L_{n-1}^{X}\left(i+m\right)-L_{n}^{X}\left(i\right)\right| \end{array}$$

where *S* is a search range. Here, $GMV^{X}\left(n\right)$ is a global motion vector along the *x* axis in the *n*-th frame.

### 2.2. Parameter Estimation

Our previous method [2] models a camera movement as a quadratic function using an initial velocity and a constant acceleration. The initial velocity and constant acceleration along the *x* direction are predicted as follows.

(3) $$\begin{array}{c} v_{n}^{X}=GMV^{X}\left(n\right)-\frac{1}{6}\left\{GMV^{X}\left(n+1\right)-2GMV^{X}\left(n\right)+GMV^{X}\left(n-1\right)\right\} \end{array}$$

(4) $$\begin{array}{c} a_{n-1}^{X}=v_{n}^{X}-v_{n-1}^{X} \end{array}$$

where $v_{n}^{X}$ and $a_{n}^{X}$ are the initial velocity and constant acceleration along the *x* direction in the *n*-th frame. The initial velocity and constant acceleration along the *y* direction are calculated in the same way. Only several numerical additions and subtractions are needed to predict compensating parameters for a single frame. Since its computational cost is small enough to be performed on low-cost reduced instruction set computer (RISC), this paper does not consider implementation of the parameter estimation on camera SoC.

### 2.3. Image Warping

In the image warping step, new images are interpolated pixel by pixel according to transformation functions as follows.

(5) $$\begin{array}{c} p_{O}\left(x,y\right)=p_{I}\left(x+x_{off}\left(y\right),y+y_{off}\left(y\right)\right) \end{array}$$

(6) $$\begin{array}{c} x_{off}\left(y\right)=c_{X}+v_{X}\times t\left(y\right)+a_{X}\times {\left(t\left(y\right)\right)}^{2} \end{array}$$

(7) $$\begin{array}{c} y_{off}\left(y\right)=c_{Y}+v_{Y}\times t\left(y\right)+a_{Y}\times {\left(t\left(y\right)\right)}^{2} \end{array}$$

Here, $p_{I}\left(x,y\right)$ and $p_{o}\left(x,y\right)$ are pixel values at (x,y) in input and output images, respectively. Offset, $\left(x_{off}\left(y\right),y_{off}\left(y\right)\right)$, is a compensation parameter. Since this method models a camera trajectory as a quadratic function, $x_{off}\left(y\right)$ and $y_{off}\left(y\right)$ in Equations (6) and (7) are quadratic functions. $v_{Y}$ and $a_{Y}$ are the initial velocity and constant acceleration along the *y* direction, respectively. $c_{X}$ and $c_{Y}$ are the initial offsets for the first row of image. t(y) is the time when capturing the *y*-th row, and the time to capture a single frame was normalized to 1 for convenience. The details are given in [2].

## 3. Proposed Method for Reducing Memory Access Amount

### 3.1. Camera SoC

#### 3.1.1. Image Processing Chain

Camera SoC includes many hardware blocks such as audio processing, image/video processing, CPU, GPIO, USB, UART, LCD out, DDR, etc. [28]. This paper considers only the image/video processing part among them. Image processing chain (IPC), which is sometimes called image processing pipeline [29], is one of the key components in image/video processing. The IPC transforms a raw image data from the CMOS sensor into a full-color image [30]. The IPC includes many image processing blocks such as dead pixel correction [31], noise reduction [32], white balance [33], demosaic [34], etc. A video encoder converts the full color images to a compressed video bitstream. The video encoder for consumer electronics usually complies with the international coding standards, such as MPEG-4 [35], H.264 [36], HEVC [37], VVC [38], etc.

**Figure 3:**
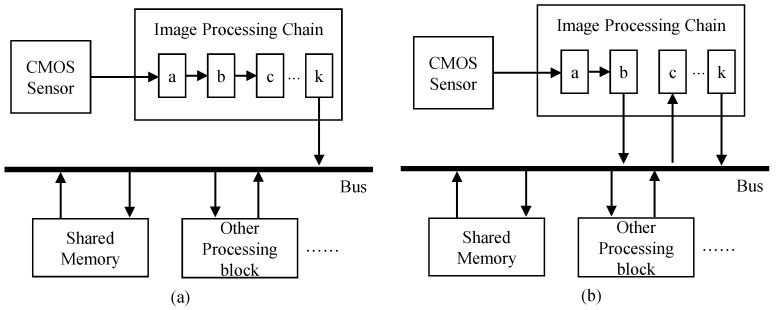
Image processing chain (**a**) without shared memory access during processing and (**b**) with shared memory access during processing.

In the view of shared memory access, the IPC can be categorized into two types. The first type of the IPC does not access the shared memory during processing as depicted in Figure 3a. The output of “*block a*” in the IPC is connected to the input of “*block b*”. Only the last processing block (or “*block k*”) accesses the shared memory to store the final image data. The remaining blocks process data on the fly without accessing the shared memory. Small buffers to temporally store several rows can be used between processing blocks. Since the output of the current processing block is fed to the input of the next processing block, the data access pattern of blocks in the IPC needs to be the same. The first block in the IPC obtains the input from the CMOS sensor that outputs raw image data in a raster scan order. Consequently, the data access patterns of all the processing blocks in the IPC need to be a raster scan order.

While many algorithms process pixels sequentially in a raster scan order, some advanced algorithms access pixels in images in a complex way. For example, some algorithms first analyze input images by accessing pixels within the images in a raster scan order, then some parts of images are selectively enhanced according to the analyzed information. Since these algorithms irregularly access pixels in images, they cannot be directly connected with processing blocks that read pixels in a raster scan order. Thus, the shared memory is required to buffer for temporally storing the intermediate frame data. Figure 3b depicts an example of the IPC with shared memory access during processing. The output of “*block b*” in the IPC is buffered in the shared memory, and “*block c*” reads input data from the shared memory.

#### 3.1.2. Requirement of VSRSC for Reducing Memory Access Usage

**Figure 4:**
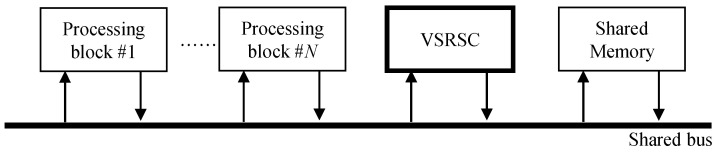
A straight method to include a video stabilization block with rolling shutter compensation in camera SoC.

The straightforward method to include VSRSC in camera SoC is simply to attach a VSRSC block as depicted in Figure 4. The VSRSC block reads frames from the shared memory via the shared bus and stores output frames to the shared memory through the shared bus. The amount of memory access used by the VSRSC block is added directly to the total memory access of camera SoC. On the other hand, since the available memory bandwidth afforded by the shared bus is limited, the reduction of required memory bandwidth is always a critical issue in camera SoC [26]. As described in the introduction, even simple VSRSC needs to read and write frames several times, which is a huge burden for camera SoC. This straightforward method, as depicted in Figure 4, is not an appropriate approach for camera SoC, where reducing memory access usage is an important issue. To eliminate the additional memory access usage caused by VSRSC, a dedicated internal memory such as SRAM may be used. However, the dedicated internal memory to store the several frames is an expensive solution, especially for low-cost devices. Hence, low-cost devices need a solution to include VSRSC by trading off performance and memory access usage.

The amount of memory access for VSRSC can be significantly reduced if a VSRSC block can be directly connected with other image processing blocks. In other words, it is desirable that the VSRSC block obtains the input data from other processing blocks and sends the output data to other blocks without accessing the shared memory. For the end, memory access patterns of the VSRSC block and other processing blocks to be connected with VSRSC should be the same. Let us consider the processing steps of VSRSC in terms of memory access. The first step is to predict motion between input frames. Motion estimation requires only input frames and generates no output data to send to other processing blocks. Thus, motion estimation can be directly connected with other processing blocks if the memory access patterns of the input of motion estimation and the output of other processing blocks are the same. The second step is to calculate parameters for compensation, which is usually performed on the CPU. Hence, there is no issue in the second step in terms of memory access. In the last step, VSRSC generates the output frame by warping the input frame based on a warping matrix. Thus, image warping can be directly connected with other processing blocks if the memory access patterns of the output of the image warping and the input of other processing blocks are the same.

#### 3.1.3. Structure of the Proposed Method for Reducing Memory Access Amount

**Figure 5:**
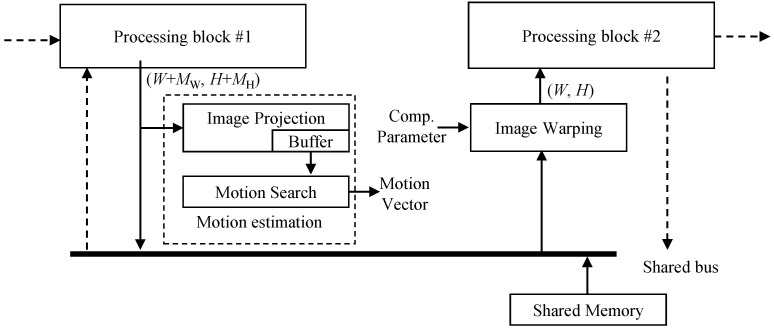
Structure of the proposed method for implementation on camera SoC.

Figure 5 depicts the structure of the proposed method. The motion estimation block in the proposed method does not directly access the shared memory through the shared bus. When processing block #1 writes data to the shared memory, the motion estimation block intercepts the data to predict motion. When processing block #2 reads data from memory, the image warping block interpolates the image on the fly, based on compensation parameter, and transfers the new pixel to the processing block #2. Therefore, this structure enables reusing data from other processing blocks #1 and #2 to predict motion and generate the output image. It should be noted here that since digital VSRSC crops input frames to generate output frames, the size of input frames should be larger than that of output frames, as shown in Figure 2b,c. For VSRSC, an image sensor captures frames larger than the final output frames. Image processing blocks located prior to the VSRSC handle the larger frames that include the processing margin of $M_{W}$ and $M_{H}$ along the horizontal and vertical directions, respectively. For instance, assume that the size of the final frame is (W,H). Then, the sizes of input frames for VSRSC will be $\left(W+M_{W},H+M_{H}\right)$. The processing block #1 should handle frames with a size of $\left(W+M_{W},H+M_{H}\right)$. Since the size of output frames from the image warp is (W,H), the processing block #2 needs to handle frames with a size of (W,H).

If the IPC accesses pixels in the shared memory during processing, such as in Figure 3b, motion estimation and image warping blocks can be located inside the IPC. The “*block b*” and “*block c*” in Figure 3b correspond to the processing block #1 and #2 in Figure 5, respectively. The motion estimation block intercepts frame data written to shared memory by “*block b*”. Image warping reads frame data from the shared memory, generates the new stabilized data, and feeds it to the “*block c*”. On the other hand, the other type of IPCs handles images without using shared memory access, as shown in Figure 3a. In this case, the motion estimation and image warping blocks are not located inside the IPC together. The motion estimation block is located at the end of the last processing block in the IPC. Thus, the processing block #1 in Figure 5 corresponds to the “*block k*” in Figure 3a. Many video cameras eventually need to encode frames processed from the IPC using video encoders. Input frames to be encoded are usually stored in the shared memory, and these video encoders read them via the shared bus. Hence, the image warping can be located at the front of the video encoder. The processing block #2 in Figure 5 corresponds to a video encoder. Some cameras do not encode frames using a video encoder. In this case of cameras, the proposed method cannot be applicable.

### 3.2. Motion Estimation

Since the blocks in the IPC process data on the fly, their pixel access pattern is the raster scan order as mentioned in Section 3.1.1. The motion estimation block is connected in the middle of the IPC or the end of the IPC. Thus, let us assume that the processing block #1 in Figure 5 outputs pixels in raster scan order.

**Figure 6:**
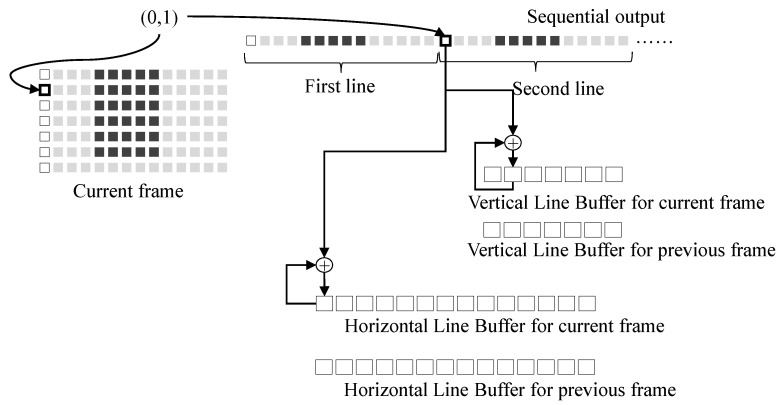
Image projection.

Motion estimation introduced in Section 2.1 consists of two steps: image projection and motion search. The first step, or image projection, is performed as follows. The processing block #1 in Figure 5 sequentially writes pixels in an image to the shared memory in a raster scan order. The data projection block in motion estimation intercepts the sequential pixels output from the processing block #1. It accumulates the sequential output to two line buffers that store two one-dimensional images along horizontal and vertical directions, respectively, as given in Equation (1). The next frames are accumulated in the same way. After line buffers for the previous and current frames are projected, the line buffers are used to perform the motion vector search according to Equation (2), which is the second step of motion estimation. Figure 6 illustrates an example of image projection in the proposed motion estimation block. Let the coordinate of the current pixel in an example of Figure 6 be (0, 1) in the current frame. The value of the current pixel is accumulated to the first element in the horizontal line buffer for the current frame and the second element in the vertical line buffer for the current frame. After all pixels in a frame are projected, a global motion estimation vector along the *x* direction is predicted using horizontal line buffers for current and previous frames according to Equation (2). A global motion vector along the *y* direction is is predicted in the same way. Two line buffers are required to store the projected lines for horizontal and vertical directions for each frame. For motion estimation, four line buffers for the previous and current frames are necessary in total. This approach makes it possible to reuse data from the other processing blocks and extremely reduces the amount of shared memory access.

It should be noted here that although the above motion estimation block predicts only global motion vectors for each frame, our previous work presents a way to calculate local line motion for each scan line from the global motion vectors [2]. The calculation of local line motion from the global motion vectors is simple enough to be performed on the low-cost RISC. Araneda presented a compact hardware architecture for motion estimation using integral projection [25]. Araneda’s work can be referenced to implement the motion estimation block of the proposed method.

### 3.3. Image Warping

In the proposed structure, the image warping block reads an image from shared memory, generates the new image, and passes the new image to the next processing block (or processing block #2) on the fly, as shown in Figure 5. The image warping needs to generate output pixels according to the memory access pattern of the next processing block. There are two types of processing blocks, as discussed in Section 3.1.1. The first type is a general image processing block, which sequentially reads pixels in an image and generates output pixels in a raster scan order. In this case, the image warp should generate output pixels sequentially in a raster scan order. The sequential output pixels from the image warp are fed to input of the next processing block. The other type of the next processing block is a video encoder where camera IPC handles images on the fly without using shared memory, as depicted in Figure 3a. The image warping should generate output pixels according to the memory access pattern of the video encoder input. The video encoders generally divide a frame into blocks and encode the divided blocks one by one. Thus, the image warp should generate output pixels based on the block. The following Section 3.3.1 and Section 3.3.2 analyze the memory access pattern, examine how to prepare output pixels in the image warp, and calculate the amount of memory access according to two types of the next processing block.

#### 3.3.1. Raster Scan Order Access

**Figure 7:**
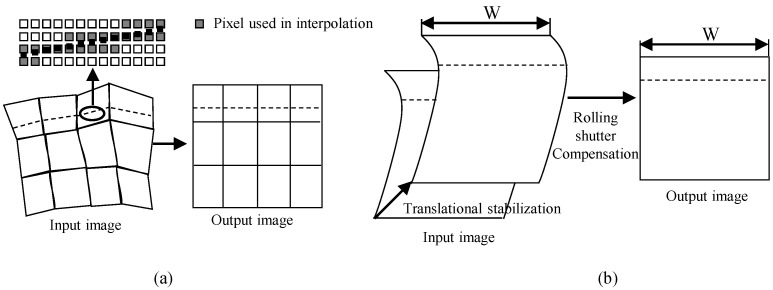
Pixel access patterns of (**a**) usual image warping and (**b**) proposed method.

In this subsection, we assume that the memory access pattern of the processing block #2 in Figure 5 is the raster scan order. VSRSC compensates camera motion and rolling shutter effect by deforming a shape of an input image to another one. Image warping deforms an input image into the new one according to the motion model with predicted model parameters. Since the state-of-the-art methods usually adopt 3D or perceptual motion models [20] to achieve high performance, the motion models are very complex, and their memory access patterns for input images become very irregular. Figure 7a illustrates one of the examples using mesh-based warping, which is generally used in conventional methods [9]. To provide output pixels in raster scan order such as the dashed line in the output image of Figure 7a, the image warping needs to predict pixel values along the dashed line in the input image. Since the positions of pixels along the dashed line are not the integer pixel accuracy, output pixels need to be interpolated using pixels in the input image. Gray pixels in the figure represent pixels to be used for interpolation. The image warps should access the gray pixels in order to generate output pixels in raster scan order. The dashed line crosses not a single row, but multiple rows in the input image. Thus, the pixel access pattern of the input image is not raster scan order. The image warp should read pixels from several rows in the input image to synthesize a single output row. The dashed line in the input image goes up and down, and memory access pattern is irregular and unpredictable. It is natural that since most conventional methods have been developed for running on personal computer, server, etc., they do not consider the memory access pattern of input images. Therefore, it is difficult to directly connect these kinds of image warping methods to a processing block that requires input pixels in a raster scan order such as in Figure 5.

Now, let us analyze the pixel access pattern of image warping for our previous work [2]. The image warp simultaneously performs video stabilization and rolling shutter compensation. Since this video stabilization adopts the translational motion model, it is performed by shifting an input image according to the predicted displacement parameters, which are with integer pixel accuracy. The input image shift can be simply performed using read memory address shift, and this video stabilization does not change the memory access pattern. This paper, hence, analyzes the pixel access pattern of only rolling shutter compensation in detail. Readout circuits in the CMOS image sensors read pixels row by row [5]. Thus, each row is captured at a different time, but pixels in the same row are captured at the same time. Pixels captured at the same time have the same amount of rolling shutter distortion, so the rolling shutter compensation parameters for pixels belonging to the same row should be the same. Pixel transformation between the input and output images is rewritten for convenience.

(8) $$\begin{array}{c} p_{O}\left(x,y\right)=p_{I}\left(x-x_{off}\left(y\right),y-y_{off}\left(y\right)\right) \end{array}$$

(9) $$\begin{array}{c} x_{off}\left(y\right)=c_{X}+v_{X}\times t\left(y\right)+a_{X}\times {\left(t\left(y\right)\right)}^{2} \end{array}$$

(10) $$\begin{array}{c} y_{off}\left(y\right)=c_{Y}+v_{Y}\times t\left(y\right)+a_{Y}\times {\left(t\left(y\right)\right)}^{2} \end{array}$$

The offset in Equation (8), or $\left(x_{off}\left(y\right),y_{off}\left(y\right)\right)$ is a function of *y*. Thus, the value of *x* component does not affect the offset value. Pixels in the same row have the same amount of offset value or compensating parameter. Accordingly, this method compensates the rolling shutter effect by moving pixels in a row in an input image to the proper position in the output image according to a compensating parameter. In other words, a horizontal line in the output image corresponds to a horizontal line in the input image with different position. Figure 7b illustrates an example of pixel access pattern for image warping for the rolling shutter compensation. To generate output pixels in raster scan order, such as the dashed line in the output image of Figure 7b, the image warping needs to access pixels along the dashed line in the input image. Since the dashed line in the input image is not slanted but horizontal, pixel access pattern is also raster scan order. Consequently, pixel access pattern of input and output pixels for image warping are the same, which satisfies the requirement described in Section 3.1.2.

**Figure 8:**
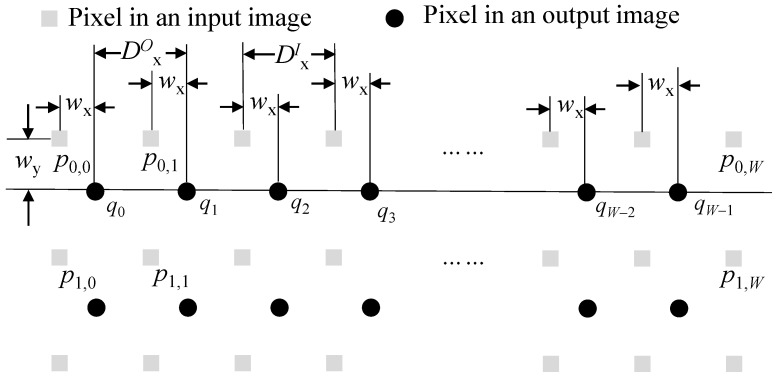
Pixels used in interpolation.

On the other hand, the offset is not integer point but float point accuracy number. Then, $(x+x_{o}ff\left(y\right),y+y_{o}ff\left(y\right)$ is a coordinate with floating point accuracy, so $p_{O}\left(x,y\right)$ needs to be obtained by interpolating pixels in the input image. For example, pixel $q_{0}$ in Figure 8 is interpolated using four pixels of $p_{0,0}$, $p_{0,1}$, $p_{1,0}$, and $p_{1,1}$. The interpolation weights for the *x* and *y* directions are $w_{x}$ and $w_{y}$, respectively. Since the offset values (or compensation parameters) of pixels from the same row are the same, the interpolation weights in the same row are constant. In an example of the figure, the interpolation weights of $q_{0}$, $q_{1}$, *…*, $q_{W-1}$ are the same along the *x* and *y* directions. Therefore, the calculation of offset and weight values only needs to be performed once for each row. For convenience of hardware implementation, the calculation can be performed in advance by CPU and be stored in internal buffers. Since the offset and weights are calculated once for each row, the computational burden is low enough to be calculated in the CPU.

**Figure 9:**
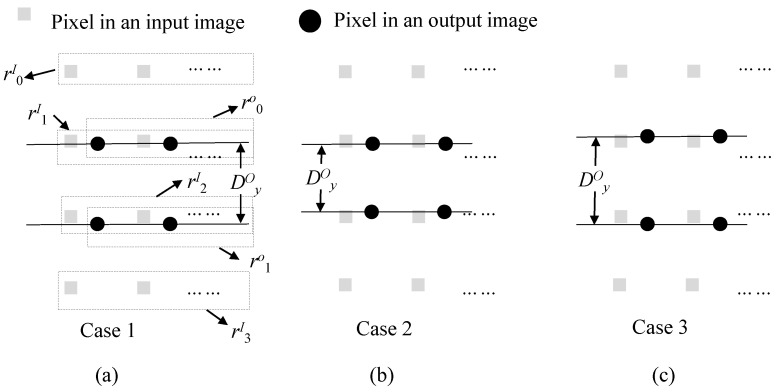
Three cases of input pixel reuse in interpolation. (**a**) One row reuse, (**b**) two rows reuse, and (**c**) no reuse.

The interval between successive output pixels along the *x* direction, or $D_{x}^{O}$ in Figure 8, is exactly 1, which is the same as $D_{x}^{I}$ or an interval between successive input pixels. Thus, the number of pixels required for interpolating one row is 2×(W+1), where *W* is the width of an output image. Some of them may be commonly used in interpolating the next row. There are three cases of input pixel reuse in interpolating the next row. The first case is a usual case in which the bottom row of two input ones used when interpolating the current output row is reused when interpolating the next output row, as depicted in Figure 9a. In the figure, two rows of $r_{1}^{I}$ and $r_{2}^{I}$ are used for interpolating pixels in a row of $r_{0}^{O}$. In addition, two rows of $r_{2}^{I}$ and $r_{3}^{I}$ are used for interpolating pixels in a row of $r_{1}^{O}$. Here, one row of $r_{2}^{I}$ is commonly used for interpolating two output rows of $r_{0}^{O}$ and $r_{1}^{O}$. Since the offset value (or $y_{off}\left(y\right)$) is varying along the *y* direction, the interval between successive output pixels along the *y* direction or $D_{y}^{O}$ in Figure 9 is not exactly 1. When the interval (or $I_{y}$) becomes less than 1, case 2 in Figure 9 sometimes occurs. In this case, two rows of $r_{1}^{I}$ and $r_{2}^{I}$ are commonly used for interpolating two output rows. The last case is where there is no common input pixels to use to interpolate two output rows, as depicted in Figure 9c. It is noted here that when the interval, $D_{y}^{O}$ is very close to 1, cases 2 and 3 are not usual.

Since two input rows are needed for interpolating one output row, the method may need to access the shared memory (2×H) times to interpolate one output image, where *H* is the height of an output image. As shown in Figure 9, some input rows are commonly reused to interpolate output rows. The amount of memory access to read input rows from the shared memory can be reduced by using a line buffer that stores and reuses the input row used in common. Under the assumption of the line buffer, $N_{access}^{T}$, the total number of rows reading from the shared memory can be calculated from the y-coordinates of rows in the input image accessed for interpolating the first and last output row. The y-coordinate of $p_{O}\left(x,y\right)$ in the input image coordinate is $\left(y-y_{off}\left(y\right)\right)$ from Equation (8). The position accessed for interpolating the first output row (or $r_{F}$ ) is $\left(0-y_{off}\left(0\right)\right)$. The position for the last output row (or $r_{L}$) is $\left(H-1\right)-y_{off}\left(H-1\right)$. Let us assume that, for simplicity, the black area [2] in CMOS image sensor is small compared to the active area, so t(H−1) is approximately 1. Note here that the time to capture a single frame was normalized to 1 for convenience. Then, $N_{access}^{T}$ is as follows.

(11) $$\begin{array}{c} N_{access}^{T}=r_{L}-r_{F}+2=\left(H+1\right)-v_{Y}-a_{Y} \end{array}$$

The amount of memory access depends on the values of $v_{Y}$ and $a_{Y}$. Camera motion along the *y* direction determines these values. For example, when there is no camera motion, $v_{Y}$ and $a_{Y}$ are zero. In this case, $N_{access}^{T}$ is (H+1). According to camera motion, $N_{access}^{T}$ can be more than or less than (H+1). If the average camera motion in a video is close to zero, the average value of $N_{access}^{T}$ at the whole frames become close to (H+1).

#### 3.3.2. Block-Based Access

Video encoders divide a frame to be encoded into small blocks and sequentially encode each block one by one. The size of the block (or basic unit) varies depending on video coding standards [35,36,37,38]. The size of macroblock for H.264 [36] is 16. The recent video coding standard, such as high-efficiency video coding (HEVC) [37], allows various sizes of the coding tree unit (CTU) from 64×64, 32×32, and 16×16. The video encoder hardware can be designed in various architectures, and the memory access pattern of reading an input block from the shared memory may be different depending on hardware architecture. This paper assumes that the video encoder hardware reads frames from a shared memory based on blocks and copies each block to the internal memory, which is one of the common methods to our best knowledge.

The image warp should provide pixels to a processing block according to a block-based pattern. In detail, the image warp reads necessary pixels in the shared memory, generates the new block using the pixels, and feeds it to the video encoder. The basic strategy is the same as that of raster scan order access described in Section 3.3.1. The difference is that the method in Section 3.3.1 uses the raster scan access in a frame level, but the block-based method uses the raster scan access in a block level. As discussed in the previous subsection, the image warp accesses more pixels than the number of output pixels to interpolate output pixels. Thus, block-based image warp requires an input block larger than k×k, in order to generate a block of k×k size to feed the next processing block. The number of lines reading from the shared memory for interpolating one block depend on the compensating parameters along the *y* direction, as discussed in the previous subsection, but we can approximate the number to $B_{H}+1$ under the assumption that the average camera motion in a video is close to zero. Here, $B_{H}$ is the block height. Then, the total memory access is as follows.

(12) $$\begin{array}{c} M_{B}^{AVG}=N_{B}\times \left(B_{W}+1\right)\times \left(B_{H}+1\right) \end{array}$$

$B_{W}$ and $N_{B}$ denote the block width and the number of blocks in a frame, respectively.

This paper assumes that the video encoder reads frames from a shared memory based on blocks and copies each block to the encoder’s internal memory. The proposed method may not be applied to encoders that do not satisfy the above assumptions.

## 4. Experimental Results and Analysis

### 4.1. Comparisons of Memory Access Amount

**Figure 10:**
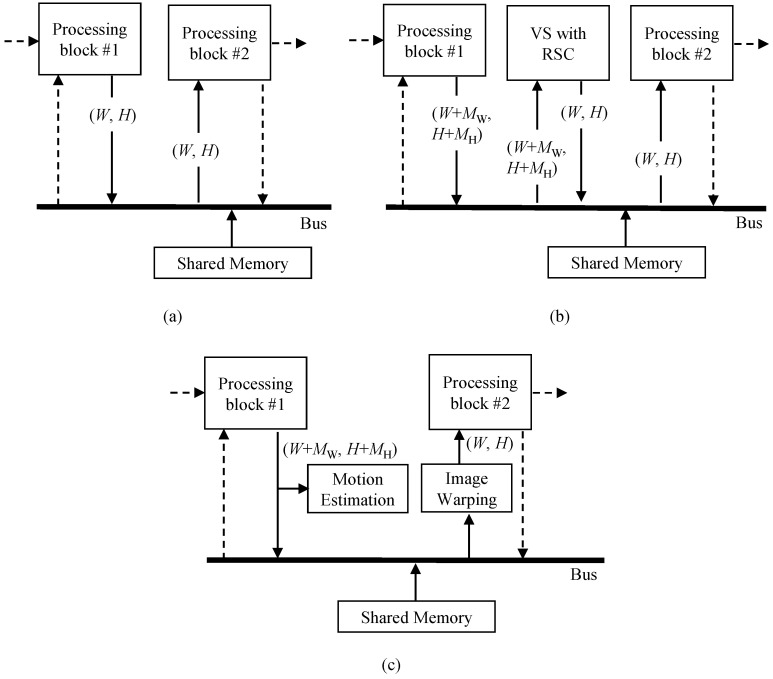
Comparison of memory access amount in three methods: (**a**) no video stabilization and rolling shutter compensation, (**b**) straightforward method, and (**c**) proposed method.

As mentioned in the introduction, few studies have been conducted to reduce the memory access amount in hardware implementation for VSRSC. Moreover, since most of the existing algorithms are based on software-based post-processing, they will require a huge amount of memory access if it is implemented with hardware. It is natural that the memory access amount of the proposed method is extremely small compared to the existing methods. Hence, the superiority of the proposed method cannot be shown by comparing the proposed method with the existing ones in terms of memory access amount. Therefore, this paper shows the effectiveness of the proposed method by comparing three algorithms. The first method does not include VSRSC in camera SoC, as given in Figure 10a. The second method considers a straightforward method to include VSRSC as shown in Figure 10b. The proposed method is applied in the final one. For fair comparisons, Figure 10b,c are assumed to adopt the same algorithm [2]. Since there are various types of camera SoCs that have many image and video processing blocks, it is not possible to examine the amount of memory access in the entire processing blocks. In this paper, we compare only the memory access amount in paths that are directly related to VSRSC. The paths correspond to the solid lines in Figure 10. The memory access paths in the dashed line are not considered. Bilinear interpolation is considered for image warping.

Without video stabilization and rolling shutter compensation, the size of output frames from the processing block #1 in Figure 10a is the same as the size of frames input to the processing block #2, where width and height are *W* and *H*, respectively. Hence, the amount of memory access to process a single frame is (2×M×N). Now, let us consider the straightforward method as shown in Figure 10b. VSRSC generally generates an output frame from an input frame by deforming and cropping the input frame, so an input frame size should be bigger than an output frame size. Let the width and height of an input image be $\left(W+M_{W}\right)$ and $\left(H+M_{H}\right)$, respectively, where $M_{W}$ and $M_{H}$ are margin for processing. The processing block #1 first writes a frame in the shared memory to be processed by VSRSC. Its memory access amount, or $B_{P}^{S}$, is $\left(W+M_{W}\right)\times \left(H+M_{H}\right)$. VSRSC then performs motion estimation and image warping. In order to predict motion between the previous and current frames, VSRSC generally needs to read the previous and current frames at least once. The amount of memory access during motion estimation, or $B_{ME}^{S}$, is $2\left(W+M_{W}\right)\times \left(H+M_{H}\right)$. For image warping, it reads an input frame from the shared memory, and synthesizes the new frame to store it to the shared memory. While the memory access amount for storing the new frame is W×H, the memory access amount for reading the input frame, $M_{R}$, is not constant. Its memory access amount, or $B_{W}^{S}$, is ($W\times H+M_{R}$). As discussed in Section 3.3.1, the amount of memory access depends on compensating parameters. The value of $M_{R}$ is proportional to $N_{access}^{T}$ in Section 3.3.1 or the total number of rows to be accessed for interpolating one output image in the image warping. If camera motion in a video is averagely close to zero along frames, the average value of $N_{access}^{T}$ of the whole frames approximates to (H+1). Hence, the value of $M_{R}$ is averagely (W+1)×(H+1), which is approximately W×H. It should be noted here that since output frames are cropped within input images, the value of $M_{R}$ cannot exceed the size of the input image with $\left(W+M_{W}\right)\times \left(H+M_{H}\right)$. Finally, the processing block #2 reads the output frame from the shared video, which amounts to W×H (or $B_{V}^{S}$). Hence, the total amount of memory access is $\left(B_{P}^{S}+B_{ME}^{S}+B_{W}^{S}+B_{V}^{S}\right)=3\left(W+M_{W}\right)\times \left(H+M_{H}\right)+2W\times H+M_{R}$, and its average and maximal values are $\left(3\left(W+M_{W}\right)\times \left(H+M_{H}\right)+3W\times H\right)$ and $\left(4\left(W+M_{W}\right)\times \left(H+M_{H}\right)+2W\times H\right)$, respectively.

In the proposed method, motion estimation is performed while processing block #1 writes output frames to the shared memory. Its memory access amounts to $\left(W+M_{W}\right)\times \left(H+M_{H}\right)$. Then, image warping reads data from the shared memory to feed its output frame to processing block #2. There are two types of processing block #2 in respect of memory access patterns. The first pattern of memory access is the raster scan order, and the other one is the block-based memory access pattern. If the memory access pattern of image warping is raster scan order, the amount of memory access becomes $M_{R}$. In this case, the total amount of memory access of the proposed method is $\left(W+M_{W}\right)\times \left(H+M_{H}\right)+M_{R}$, and its maximal value is $2\left(W+M_{W}\right)\times \left(H+M_{H}\right)$. The second access pattern is block-based memory access. The average amount of memory access, or $M_{B}^{AVG}$, is given in Equation (12), and the total amount of memory access is $\left(W+M_{W}\right)\times \left(H+M_{H}\right)+M_{B}^{AVG}$. Since $M_{B}$ is $W\times H/(B_{W}B_{H})$, Equation (12) can be rewritten as

(13) $$\begin{array}{c} M_{B}^{AVG}=W\times H\times \left(1+B_{W}^{-1}+B_{H}^{-1}+B_{W}^{-1}B_{H}^{-1}\right) \end{array}$$

Table 1 summarizes the memory access amount required for processing a single frame. The proposed method significantly reduces the amount of memory access compared to the straightforward method. The additional memory access amount by the proposed method is extremely small compared to no VSRSC. Table 2 shows the memory access amount of a typical example. It is assumed here that the frame size is 1920×1080 and the margin is 10% for the *x* and *y* directions. In the table, the straightforward method averagely increases the memory access amount by 232%, in order to support VSRSC. Meanwhile, the proposed method averagely increases only 11% of the memory access amount. The proposed one reduces the memory access amount by 21.1 times (=232%/11%) compared to the straightforward method.

The total amounts of memory accesses for no VSRSC and the proposed method (raster scan order) are averagely (2×M×N) and $\left(\left(W+M_{W}\right)\times \left(H+M_{H}\right)+M\times N\right)$, respectively. The additional memory access amount of the proposed method becomes $\left(M_{W}\times H+M_{H}\times W+M_{W}\times M_{H}\right)$. Let the margins for the *x* and *y* directions be $\alpha_{W}$ and $\alpha_{H}$ in terms of percentage. Then, the increase rate of additional memory access amount is as follows.

(14) $$\begin{array}{c} \frac{M_{W}H+M_{H}W+M_{W}M_{H}}{2WH}=0.5\left(\alpha_{W}+\alpha_{H}+\alpha_{W}\alpha_{H}\right) \end{array}$$

where $M_{W}=\alpha_{W}W$ and $M_{H}=\alpha_{H}H$. Hence, the increased rate of additional memory access amount is proportional to the sum of margin rates along the *x* and *y* directions by approximating $\alpha_{W}\alpha_{H}<<\alpha_{W}$ and $\alpha_{W}\alpha_{H}<<\alpha_{H}$. The strength of this work is that it performs VSRSC with extremely small increase of memory access.

The margin area is required to perform VSRSC. Accordingly, IPC should handle an image larger than the final output image, which increases the implementation cost of the IPC. The IPC needs to additionally process data of size $\left(\left(W+M_{W}\right)\times \left(H+M_{H}\right)-W\times H)\right)$. If the size of the final output image is 1920×1080 and the margin is 10% for the *x* and *y* directions, the amount of data to be additionally processed by the IPC is 21%.

### 4.2. Image Quality

Video stabilization techniques have been studied extensively over the past decade. Recent algorithms show high performance in terms of video quality, but, as mentioned in the previous chapter, most algorithms are software-based post-processing and use a large amount of computation. On the other hand, consumer cameras use fast real-time algorithms or mechanical optical image stabilization. As these fast algorithms have been developed for real-time operation, the performance is inferior to that of the software-based post-processing algorithm. Since the proposed algorithm is also designed for the real-time processing, its performance is also inferior to that of the software-based post-processing algorithm.

The performance gap between the proposed method and the state-of-the-art techniques depends on the type of input videos. Since the proposed algorithm adopts a 2D-based motion model, the performance gap is very small for videos having small camera motion. However, as the camera motion becomes large, the 2D-based motion model will not accurately model the camera motion, and its performance will degrade. A trade-off between performance and computation is inevitable in real-time application. The performance of the proposed method is the same as that of our previous algorithm. The detailed performance comparisons are given in [2].

Although the proposed method is not superior to the state-of-the-art methods applied in post-processing in terms of video quality, it provides sufficient performance to lower the cost of camera hardware for the development of real-time devices. It requires only an additional 11% increase of memory access on average for video stabilization and rolling shutter compensation when the margin is 10%. Therefore, it is very suitable for low-cost camera hardware.

### 4.3. Extra Processing Delay

Motion estimation of the proposed method consists of two parts: data projection and motion search. As discussed in Section 3.2, the data projection needs only simple operations. Hence, the extra processing delay is not an issue in data projection. The motion search starts after finishing the data projection of the current frame. In order not to increase the processing delay, this motion search should be completed before starting the projection of the next frame. There is a black area [2] in CMOS image sensor where it is a non-image area. Since the motion search in the proposed method is a one-dimensional search problem, it may be possible to perform within this non-image timing. If the motion search cannot be completed within the black area time, three line buffers are required for triple buffering. In this case, the processing delay increases by one frame.

The interpolation weight for the same row is constant, as discussed in Section 3.3. The calculation of interpolation weight only needs to be performed once for each row. In addition, interpolation requires only several numerical operations. Hence, the processing delay of the image warping is not a problem.

## 5. Conclusions

Video stabilization with rolling shutter compensation generally requires high computational burden and huge memory access amount. Existing research focused on the development of software-based post processing algorithms, and it was difficult to apply these algorithms to low-cost cameras with limited hardware resources. Some fast algorithms were developed in order to alleviate the computational burden by trading off between performance and processing speed, but the memory access amount required to perform video stabilization with rolling shutter compensation is still too huge to run on low-cost camera hardware. This work presents a structure and layout method to efficiently implement our previous real-time algorithm for low-end camera hardware in terms of shared memory access amount. The proposed method places sub-components in a parasitic form in other processing blocks, and the sub-components reuse data read from other processing blocks. Hence, the additional memory access amount will be extremely small. The proposed method thus has high application potential, particularly in low-cost devices.

VSRSC consists of some sub-components. The proposed method places each sub-component in a parasitic form in other processing blocks to reduce the memory access. Hence, all processing blocks in the camera SoC related to the VSRSC should be implemented in hardware to run the proposed method in real time, which requires very high cost. This makes it difficult to evaluate the real-time performance of the proposed method at this time. Further work in the real-time implementation on camera SoC needs to be carried out to evaluate the proposed method.

## Figures and Tables

**Table 1:** Memory access amount required for processing a single frame. Here, VSRSC stands for video stabilization with rolling shutter compensation.

Method		Memory Bandwidth
No VSRSC		2×M×N
Straightforward method	On average	$3\left(W+M_{W}\right)\times \left(H+M_{H}\right)+3W\times H$
	maximum	$4\left(W+M_{W}\right)\times \left(H+M_{H}\right)+2W\times H$
Proposed method Raster scan order	On average	$\left(W+M_{W}\right)\times \left(H+M_{H}\right)+W\times H$
	maximum	$2\left(W+M_{W}\right)\times \left(H+M_{H}\right)$
Proposed method Block-based (on average)		$\left(W+M_{W}\right)\times \left(H+M_{H}\right)+M_{B}^{AVG}$

**Table 2:** Memory access amount for a frame with a size of 1920×1080. Let the block size for the video encoder be 64. The margin size is set to 10%.

Method		Memory Bandwidth	
		Value	Ratio
No VSRSC		4,147,200	1.00
Straightforward method	On average	13,747,968	3.32
	maximum	14,183,424	3.42
Proposed method raster scan order	On average	4,582,656	1.11
	maximum	5,018,112	1.21
Proposed method Block-based (on average)		4,778,752	1.15
